# Effects of *Eimeria tenella* Infection on Key Parameters for Feed Efficiency in Broiler Chickens

**DOI:** 10.3390/ani11123428

**Published:** 2021-12-01

**Authors:** Janghan Choi, Hanseo Ko, Yuguo Hou Tompkins, Po-Yun Teng, Jeferson M. Lourenco, Todd R. Callaway, Woo Kyun Kim

**Affiliations:** 1 Department of Poultry Science, University of Georgia, Athens, GA 30602, USA; choij@uga.edu (J.C.); hsko@uga.edu (H.K.); yuguot@uga.edu (Y.H.T.); pyteng@uga.edu (P.-Y.T.); 2 Department of Animal and Dairy Science, University of Georgia, Athens, GA 30602, USA; jefao@uga.edu (J.M.L.); todd.callaway@uga.edu (T.R.C.)

**Keywords:** *Eimeria tenella*, broiler chickens, oocyst shedding, volatile fatty acids, feed efficiency, cecal health

## Abstract

**Simple Summary:**

Coccidiosis, which can be induced by *Eimeria* spp., causes tremendous economic losses in the poultry production. *Eimeria tenella* (*E. tenella*) is one of the poultry *Eimeria* spp. that damage cecal tissue. Broilers infected with *E. tenella* can have reduced body weight, feed efficiency, and gut health because ceca are the main site for producing volatile fatty acids (VFA; important energy sources) and ceca accommodate diverse pathogens. To find appropriate strategies to cope with *E. tenella* infection, modes of actions of *E. tenella* infection on broiler growth and health should be investigated, and experimental infection model should be established. In the study, different levels of sporulated *E. tenella* oocysts were inoculated to the broilers, and the inoculation dosages induced mild infection in the ceca of broilers. The current study showed that *E. tenella* infection damaged feed efficiency and small intestinal health in broilers, mainly by reducing cecal volatile fatty acids (VFA) production. Different inoculation levels modulated the tendency of fecal moisture content and fecal oocyst shedding at different time points. Based on the results, energy supplementation and/or modulation of cecal microbiota potentially ameliorates negative effects of *E. tenella* infection in broilers.

**Abstract:**

The purpose of the study was to investigate effects of different inoculation dosages of *E. tenella* on growth performance, gastrointestinal permeability, oocyst shedding, intestinal morphology, fecal consistency, ileal apparent digestibility, antioxidant capacity, and cecal VFA profile in broiler chickens. Five different dosages (T0: 0, T1: 6250, T2: 12,500, T3: 25,000, and T4: 50,000) of *E. tenella* oocysts were inoculated via oral gavage to fourteen-day-old broilers. Inoculation of *E. tenella* linearly increased FCR (*p* < 0.05), and feed intake was quadratically increased on 6 days post-infection (dpi; *p* = 0.08) and 7 dpi (*p* = 0.09). Cecal lesion score of each treatment was T0: 0; T1: 0.39 ± 0.14; T2: 0.93 ± 0.21; T3: 1.25 ± 0.16; and T4: 1.58 ± 0.2. Cecal total VFA production was linearly reduced due to *E. tenella* infection on 6 dpi (*p* < 0.01). *E. tenella* infection deepened cecal crypts depth on 6 dpi (CD; *p* < 0.05). Gastrointestinal permeability tended to be linearly increased (*p* = 0.07). *E. tenella* infection tended to linearly reduce duodenal VH (*p* = 0.1) and jejunal VH on 9 dpi (*p* = 0.09). Different dosages of *E. tenella* modulated the tendency of fecal moisture content and oocyst shedding. Therefore, *E. tenella* infection impaired feed efficiency and small intestinal health mainly by reducing cecal VFA production and deepening cecal CD in broilers.

## 1. Introduction

Coccidiosis causes tremendous economic losses in broiler production by impairing gut health and depressing growth performance and feed efficiency of broiler chickens, and expensive anti-coccidial treatments also increase the overall production cost [1,2]. Avian coccidiosis are induced by *Eimeria* spp., which are protozoan parasites, and there are 7 known *Eimeria* spp. that can infect chickens: *Eimeria acervulina*, *E. maxima*, *E. tenella*, *E. brunetti*, *E. necatrix*, *E. mitis,* and *E. praecox* [3]. Each species resides at the different section of the intestinal tract of broiler chickens, and thereby it has different modes of actions to affect growth performance and gut health of broilers [4]. Teng et al. [5] reported that *E. maxima* decreased digestibility of crude proteins and amino acids in broilers. *Eimeria* spp. can be transmitted via the fecal–oral route. The infection is initiated by ingestion of sporulated (infectious) oocysts, and after the asexual and sexual replications, un-sporulated oocysts are excreted with feces [6]. In an appropriate environment, the oocysts can be sporulated and become infectious, and this life cycle can be repeated with poultry growth cycle.

*Eimeria tenella* (*E. tenella*) resides in the mucus membrane of ceca, and during its replications, epithelial cells in ceca are damaged, resulting in hemorrhagic diarrhea and impaired growth performance and intestinal health in broilers [7]. The ceca, the main intestinal compartment for bacterial fermentation, can be reservoirs for pathogenic bacteria and their toxins that can cause oxidative stress after entering the blood stream of broilers [8]. However, ceca also play crucial roles in producing beneficial bacterial metabolites including vitamins, volatile fatty acids (VFA), lactic acid, and antimicrobial compounds via bacterial fermentation [9]. The VFA are not only inhibit the growth of pathogenic bacteria, but also are energy substrates for the host and induce gut development of chickens by accelerating gut epithelial cell proliferation [10]. Moreover, VFA interact with fat metabolism via mitogen-activated protein kinase (MAPK) pathway [11]. These suggest that VFA are closely associated with feed efficiency by providing extra energy to the host or influencing metabolism of chickens.

To cope with *Eimeria* spp. infection in broiler production, anti-coccidial drugs, and vaccination has been used in the broiler industry. However, the use of anti-coccidial drugs has been restricted by inhibiting the use of old anti-coccidial drugs and requiring Veterinary Feed Directives (VFD) registrations because of the spread of resistant *Eimeria* strain and consumer pressure [12,13]. Furthermore, vaccination is expensive and can prevent spread of *Eimeria* spp. [14]. Recently, a lot of attention has been paid to find nutritional interventions to control *Eimeria* spp. infection in broilers. Diverse bioactive compounds, including essential oils [15], probiotics [16], sodium butyrate [17], and plant extracts [18], were studied to control or to ameliorate negative effects of *E. tenella* infection and in broilers. The modes of actions of those bioactive compounds may include damaging cell wall of *E. tenella*, modulating cecal microbiota, and/or enhancing the immunity of broilers. To find suitable nutritional interventions, it is important to understand mode of actions of *E. tenella* on the growth of chickens and to set up appropriate experimental infection models to test novel nutritional interventions. Therefore, the hypothesis of this study was that impaired cecal health due to *E. tenella* infection may result in reduced growth performance and impaired intestinal health because of reduced VFA production and increased oxidative stress. The purpose of the study was to investigate the effects of different inoculation dosages of *E. tenella* on growth performance, gastrointestinal permeability, oocyst shedding, fecal consistency, intestinal morphology, ileal apparent digestibility, antioxidant capacity, and cecal VFA in broiler chickens.

## 2. Materials and Methods

### 2.1. Experimental Design, Diets, and Growth Performance

This study was approved by the Institutional Animal Care and Use committee of the University of Georgia, and this experiment was conducted at the Poultry Research Center, University of Georgia, Athens, GA. A total of 360 fourteen-day-old male Cobb500 broiler chickens were distributed to 5 treatments with 6 replicates (12 birds per battery cage) in a completely randomized design. The experimental treatments were (1) treatments 0 (T0): administration with 1 mL of phosphate-buffered solution (PBS) as a sham-challenged group; (2) treatments 1 (T1): administration with 6250 sporulated oocysts of *E. tenella*; treatment 2 (T2): administration with 12,500 sporulated oocysts of *E. tenella*; treatments (T3): administration with 25,000 sporulated oocysts of *E. tenella*; and treatment (T4), administration with 50,000 sporulated oocysts of *E. tenella*. The sham-challenged groups were placed on the top of the cages to minimize the cross-infection. The *E. tenella* used in the study was a wild-type strain. To each bird, 1 mL of inoculum was administrated by oral gavage. As shown in Table 1, the experimental diet (D 14 to 23) was formulated to meet or exceed Cobb 500 nutrient requirements (2018) and included 3 g/kg of titanium (IV) oxide (Acros Organics, Morris Plains, NJ, USA) as an indigestible marker to determine the apparent ileal digestibility (AID). In-feed anticoccidials were not included in the experimental diet (D 14 to 23) and in the pre-experimental diet (D 0 to 14).

During the entire experiment period, birds had free access to water and feed, and temperature was controlled according to the recommendation of Cobb Broiler Management Guide. Body weight (BW) of the birds per cage were recorded on 6 days post-infection (dpi) and 9 dpi, and feed disappearance were recorded daily to calculate average daily gain (ADG), average daily feed intake (ADFI), and feed conversion ratio (FCR). The acute phase was 0 to 6 dpi, and recovery phase was 6 to 9 dpi.

### 2.2. Gastrointestinal Permeability, Oocyst Shedding, and Fecal Consistency

Gastrointestinal permeability was measured according to Teng et al. [19] with minor modifications. On 5, 6, and 7 dpi, one bird per cage was administrated with 1 mL of 2.2 mg/mL of fluorescein isothiocyanatedextran 4 kDa (FITC-D4; Sigma-Aldrich Co., St. Louis, MO, USA) dissolved in PBS. After 2 h, birds were euthanized and blood from the heart was collected into heparin-free vacutainer tubes. The tubes stood in a dark container at room temperature for 1 h for clotting and centrifuged at 1000× *g* for 15 min to recover serum. The collected serum samples were transferred to a 96 black well plate (Greiner Bio-one, Monroe, NC, USA) in duplicate, and the fluorescence was measured at an excitation wavelength of 485 nm and an emission wavelength of 528 nm by using a ICTOR3 Multilabel Plate Reader (Perkin Elmer, Waltham, MA, USA). The FITC-D4 concentration in the serum was quantified by prepared standard solution using pooled serum from birds that were not inoculated with FITC-D4 (not part of the study) and expressed relative to the T0 group.

Fresh fecal samples were collected on 5 to 6 dpi, 6 to 7 dpi, and 7 to 8 dpi to measure fecal moisture content and fecal oocyst shedding. Fecal samples were put in a 60 °C oven until constant weight, and the weights before and after drying were recorded to calculate moisture content. Oocysts in fecal samples were counted using a McMaster chamber to calculate oocyst shedding per gram of feces. Briefly, 3 to 5 g of fecal samples were put in 50 mL tubes and mixed thoroughly with 25 mL of distilled water to ensure a uniform suspension. Afterwards, 1 mL of the suspension were mixed with 9 mL of the saturated salt solutions. The mixed solution was loaded into a McMaster chamber, and number of oocysts were counted using a microscope. Oocyst shedding were expressed as log_10_ (oocysts/g feces).

### 2.3. Sample Collection and Lesion Score

On 6 and 9 dpi, 4 birds per cage were euthanized by the cervical dislocation method for sample collection and lesion scoring (6 dpi). Cecal lesion scoring from 4 birds per cage was conducted in a blind fashion according to the 4-score scale method [20]. Around 3 to 5 cm of intestinal sections of mid-duodenum, mid-jejunum, mid-ileum, and mid-ceca were collected, and then rinsed with PBS to remove digesta, and stored in 10% neutral-buffered formalin for further steps. From 4 birds per cage, ileal digesta (from the Meckel’s diverticulum to 15 cm upper from the ileo-cecal-colic junction) were collected and dried in a 60 °C oven until constant weight to determine ileal moisture content and for digestibility analysis. Liver and ceca samples were collected and snap-frozen and stored at −80 °C.

### 2.4. Intestinal Morphology

The fixed tissues in 10% neutral-buffered formalin were embedded in paraffin and cut into 4 µm, and hematoxylin and eosin (H&E) staining was conducted. The H&E-stained slides were read using a microscope (BZ-X810; Keyence, Osaka, Japan). The villus height (VH) and crypts depth (CD) of five well-oriented villi per section and their corresponding crypts for the five villi were measured for duodenum, jejunum, and ileum samples, and CD was measured for ceca samples by using ImageJ (National Institutes of Health, Bethesda, MD, USA). The ratios of VH to CD were calculated for each villus and crypt.

### 2.5. Apparent Ileal Digestibility

The concentrations of titanium dioxide in oven-dried samples (0.3 g for ileal digesta samples and 0.5 g for the feed sample) were analyzed according to Short et al. [21]. Dry matter (DM), organic matter (OM), and ash apparent ileal digestibility were determined according to Lin and Olukosi [22].

### 2.6. Liver Total Antioxidant Capacity (TAC)

Total antioxidant capacity (TAC) in the liver was measured using a commercial kit (QuantiCromAntioxidant Assay Kit; DTAC-100) (BioAssay Systems, Hayward, CA, USA). Approximately 100 mg of frozen liver samples were homogenized in 1 mL of PBS for 45 s using a beads beater and centrifuged at 10,000× *g* for 10 min. Aliquots of supernatants were taken for the analyses of protein content using the Pierce™ BCA Protein Assay Kit (Thermo Fisher Scientific, Cleveland, OH, USA) after 20 times dilution. Afterwards, TAC was measured according to the manufacture’s protocol without further dilutions. The absorbance was measured using SpectraMax^®^ ABS Plus microplate reader (Molecular devices, San Jose, CA, USA). The TAC values were expressed as nM trolox quivalents/mg protein.

### 2.7. VFA Concentrations in Cecal Digesta

Concentrations of VFA in cecal digesta were analyzed according to Lourenco et al. [23]. Cecal samples were collected from birds on 6 and 9 dpi, and the samples were snap-frozen in liquid nitrogen and stored at −80 °C for further analyzes. Once thawed, samples were diluted and homogenized by placing 0.5 g into 3 mL of distilled water. The samples were vigorously homogenized for 1 min and subsequently frozen at −20 °C. After the samples were thawed, these were centrifuged at 10,000× *g* for 10 min, and 850 μL of supernatant were collected and mixed with 170 μL of the fresh 25% (wt/vol) meta-phosphoric acid solution, and immediately frozen at −20 °C overnight. The samples were centrifuged at 10,000× *g* for 10 min, and 800 μL of supernatant was collected and mixed with 1600 μL ethyl acetate. Samples were vigorously homogenized for 10 s and allowed to settle for 5 min. The top layer was transferred to a screw-thread vial and analyzed in a gas chromatograph (Shimadzu GC-2010 plus; Shimadzu Corporation, Kyoto, Japan) equipped with an autoinjector (AOC-20i; Shimadzu Corporation, Kyoto, Japan). A capillary column (Zebron ZB-FFAP; 30 m × 0.32 mm × 0.25 μm; Phenomenex Inc., Torrance, CA, USA) was used for the separation of the VFA. The sample injection volume was set at 1 mL, and helium was used as the carrier gas. The column temperature was initially set at 110 °C, and gradually increased to 200 °C over the course of 6 min. The flame ionization detector was set at 350 °C.

### 2.8. Statistical Analyses

Statistical analyses were performed using SAS (version 9.4; SAS Inst. Inc., Cary, NC, USA). Data normality was checked using proc univariate except for lesion score data. All groups were compared using proc mixed in a completely randomized design followed by Tukey’s comparison test. Kruskal–Wallis test followed by the Dwass, Steel, Critchlow-Fligner post hoc test was used to analyze lesion score data. Orthogonal polynomial contrasts were utilized to evaluate the significance of linear or quadratic effects of different *E. tenella* inoculation dosages, and the inoculation dosages of *E. tenella* were normalized by using the base 2 logarithm of the number of sporulated *E. tenella* number for orthogonal polynomial contrasts [19]. Statistical significance was set at *p* < 0.05, and trends (0.05 ≤ *p* ≤ 0.1) were also presented. 

## 3. Results

### 3.1. Growth Performance and Lesion Score

**Figure 1:**
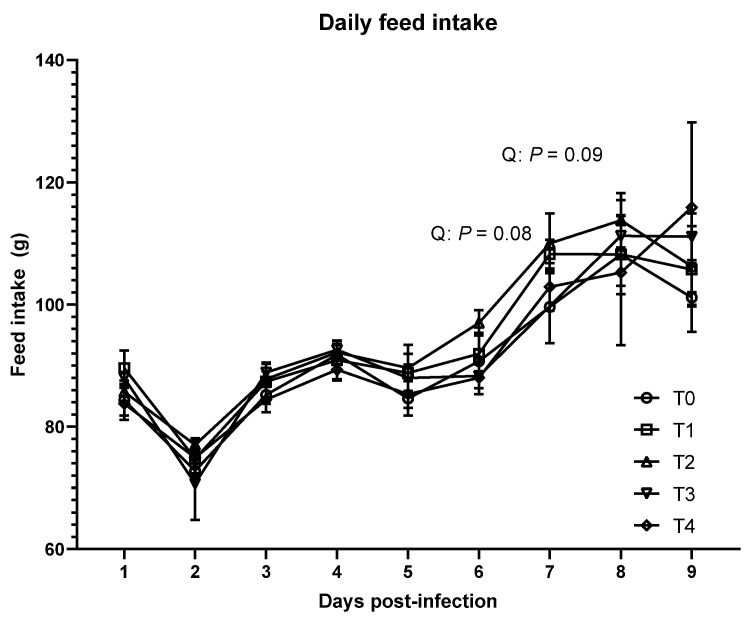
Daily feed intake of broiler chickens infected with different dosages of *Eimeria tenella*. Daily feed intake was measured in the T0 (treatment 0; Sham-challenged with phosphate-buffered saline); T1 (treatment 1; challenged with 6250 sporulated oocysts of *E. tenella*); T2 (treatment 2; challenged with 12,500 sporulated oocysts of *E. tenella*); T3 (treatment 3; challenged with 25,000 sporulated oocysts of *E. tenella*); T4 (treatment 4; challenged with 50,000 sporulated oocysts of *E. tenella*) groups during 1 to 9 days post-infection. At each time point, orthogonal polynomial contrasts analysis was conducted to see linear pattern (L) and quadratic pattern (Q) among the treatments.

As shown in Table 2, no significant differences were observed in BW, ADG, and ADFI in the acute phase (*p >* 0.1) among the treatments. However, daily feed intake tended to quadratically increase on 6 dpi (*p* = 0.08) and 7 dpi (*p* = 0.09) due to *E. tenella* infection (Figure 1). In the acute phase, FCR was linearly increased due to the inoculation of *E. tenella* (*p* < 0.05). There were no significant differences in growth performance among the treatments in the recovery phase.

**Figure 2:**
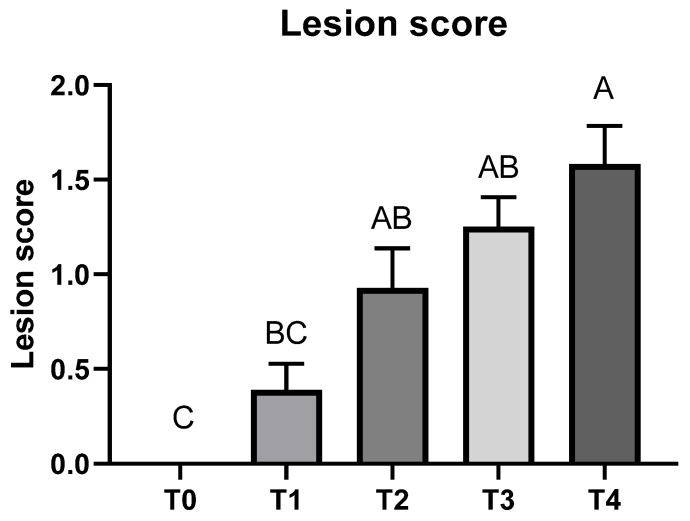
Cecal lesion score of broiler chickens infected with different dosages of *Eimeria tenella* on 6 days post-infection. Cecal lesion score was measured in the T0 (treatment 0; Sham-challenged with phosphate-buffered saline); T1 (treatment 1; challenged with 6250 sporulated oocysts of *E. tenella*); T2 (treatment 2; challenged with 12,500 sporulated oocysts of *E. tenella*); T3 (treatment 3; challenged with 25,000 sporulated oocysts of *E. tenella*); T4 (treatment 4; challenged with 50,000 sporulated oocysts of *E. tenella*) groups on 6 days post-infection. Different letters at the same time point represent significantly different (*p* < 0.05) by utilizing the Kruskal–Wallis test followed by the Dwass, Steel, Critchlow-Fligner post hoc test.

Cecal lesion due to *E. tenella* infection was not detected in the T0 group on 6 dpi (Figure 2). The T4 group had a higher lesion score compared to the T1 group (*p* < 0.05); lesion scores > 0 and <2 were also obtained in T1, T2, and T3 groups.

### 3.2. Oocyst Shedding and Fecal/ileal Moisture Content

**Figure 3:**
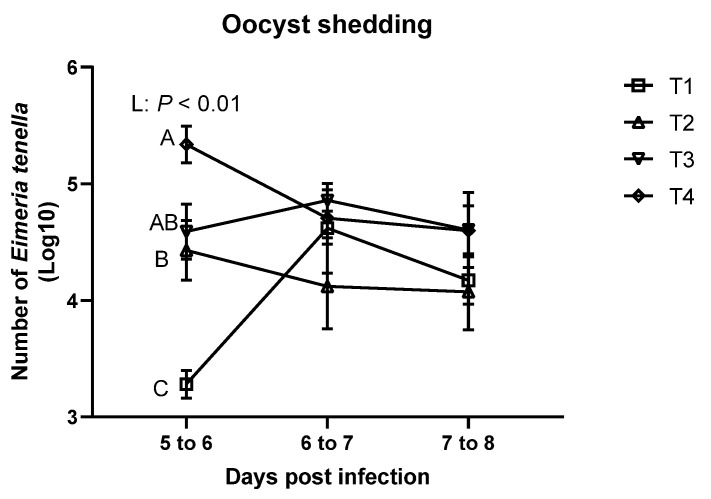
Oocyst shedding of broiler chickens infected with different dosages of *Eimeria tenella*. Fecal *oocyst shedding* was measured using McMaster chamber in the T0 (treatment 0; Sham-challenged with phosphate-buffered saline); T1 (treatment 1; challenged with 6250 sporulated oocysts of *E. tenella*); T2 (treatment 2; challenged with 12,500 sporulated oocysts of *E. tenella*); T3 (treatment 3; challenged with 25,000 sporulated oocysts of *E. tenella*); T4 (treatment 4; challenged with 50,000 sporulated oocysts of *E. tenella*) groups on 5 to 6, 6 to 7, and 7 to 8 days post-infection. Number of *E. tenella* oocysts is shown as log_10_ (oocysts/g feces). At each time point, orthogonal polynomial contrasts analysis was conducted to see linear pattern (L) and quadratic pattern (Q) among the treatments. Different letters at the same time point represent significantly different (*p* < 0.05) by proc mixed followed by the Tukey’s multiple comparison test among the treatment groups.

As shown in Figure 3, *E. tenella* was not detected in the feces of the sham-challenged (T0) group at all time points. On 5 to 6 dpi, the T4 group had significantly higher oocyst shedding compared to T1 group, and *E. tenella* infection linearly increased oocyst shedding. However, there were no significant differences in oocyst shedding among the treatments on 6 to 7 dpi and 7 to 8 dpi.

As shown in Table 3, Fecal moisture content was modulated due to *E. tenella* infection on 7 to 8 dpi (*p* < 0.05). Fecal moisture content was quadratically decreased on 5 to 6 dpi (*p* < 0.05), and quadratically increased on 6 to 7 dpi (*p* < 0.05) due to *E. tenella* infection. *E. tenella* infection linearly increased fecal moisture content on 7 to 8 dpi (*p* < 0.05). On 6 dpi, *E. tenella* infection altered ileal moisture content, and ileal moisture content was linearly decreased on 6 dpi due to *E. tenella* infection (*p* < 0.05).

### 3.3. Gastrointestinal Permeability

**Figure 4:**
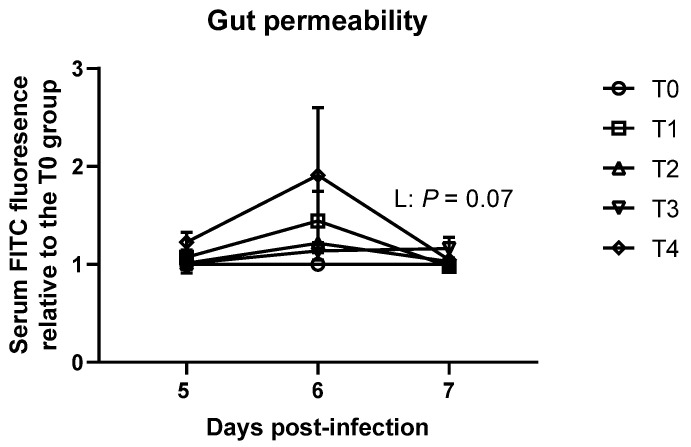
In vivo gastrointestinal permeability of broiler chickens infected with different dosages of *Eimeria tenella*. In vivo gastrointestinal permeability was measured using fluorescein isothiocyanate dextran 4 kDa in the T0 (treatment 0; Sham-challenged with phosphate-buffered saline); T1 (treatment 1; challenged with 6250 sporulated oocysts of E. tenella); T2 (treatment 2; challenged with 12,500 sporulated oocysts of *E. tenella*); T3 (treatment 3; challenged with 25,000 sporulated oocysts of *E. tenella*); T4 (treatment 4; challenged with 50,000 sporulated oocysts of *E. tenella*) groups on 5, 6, and 7 days post-infection. At each time point, orthogonal polynomial contrasts analysis was conducted to see linear pattern (L) and quadratic pattern (Q) among the treatments.

No significant differences in gastrointestinal permeability were observed on 5 dpi and 6 dpi (Figure 4). However, gastrointestinal permeability tended to be linearly increased (*p* = 0.07). However, the numerically highest group, T4, had only 1.16 folds FITC fluorescence compared to the T0 group.

### 3.4. Intestinal Morphology and Apparent Ileal Digestibility

**Figure 5:**
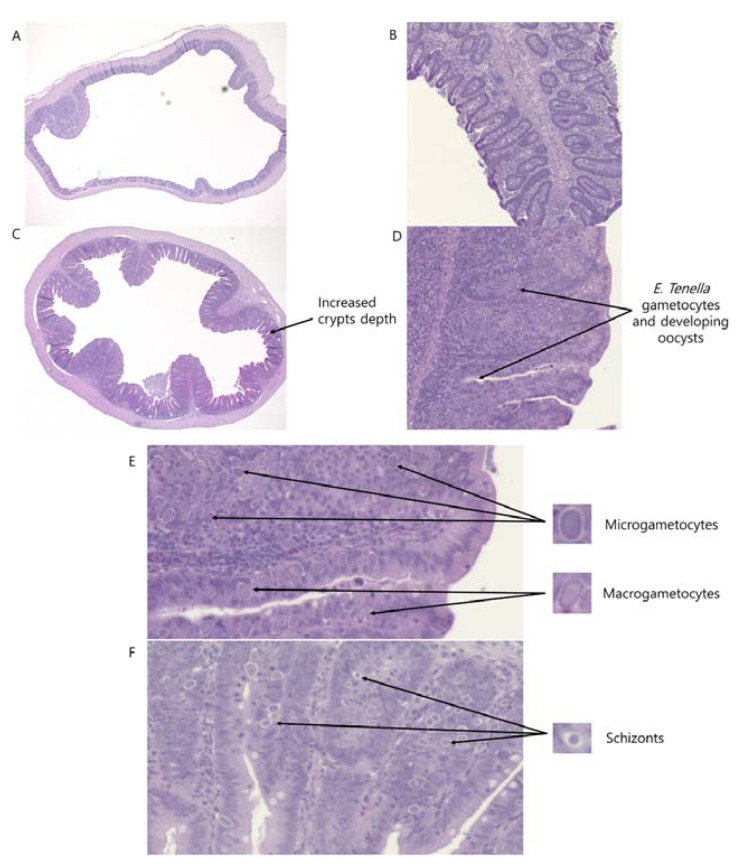
Ceca morphology of a non-challenged bird ((**A**) 2×; (**B**) 10×) and of a *Eimeria tenella* infected bird ((**C**) 2×; (**D**) 10×) in the T1 group (challenged with 6250 sporulated oocysts of *E. tenella*) on 6 days post-infection when tissues were stained with hematoxylin and eosin. Microgametocytes, macrogametocytes, and schizonts were observed in the ceca crypts (**E**,**F**).

As shown in Table 4, *E. tenella* infection linearly (*p* < 0.05) and quadratically (*p* < 0.05) increased cecal CD, whereas the sham-challenged group had the lowest cecal CD compared to the *E. tenella* challenged groups on 6 dpi (*p* < 0.05). On 6 dpi, ileal CD showed a tendency to be linearly reduced (*p* = 0.06), and ileal VH:CD tended to be increased (*p* = 0.08) due to *E. tenella* infection. On 9 dpi, the inoculation of *E. tenella* tended to linearly decrease duodenal VH (*p* = 0.1) and jejunal VH (*p* = 0.09). The T0 group had lower cecal CD compared to T2, T3, and T4 groups (*p* < 0.05), and *E. tenella* infection linearly (*p* < 0.05), and quadratically (*p* < 0.05) increased cecal CD. As shown in Figure 5, cecal CD were deepened, and gametocytes and developing oocysts were observed in the ceca of broiler chickens infected *E. tenella.*

In broiler chickens infected *E. tenella*, crypts were deepened and gametocytes and developing oocysts were observed.

There were no significant differences in the ileal apparent digestibility of DM, OM, and ash among treatments on 6 dpi and 9 dpi (*p* > 0.1; Table 5).

### 3.5. Liver Total Antioxidant Capacity (TAC)

**Figure 6:**
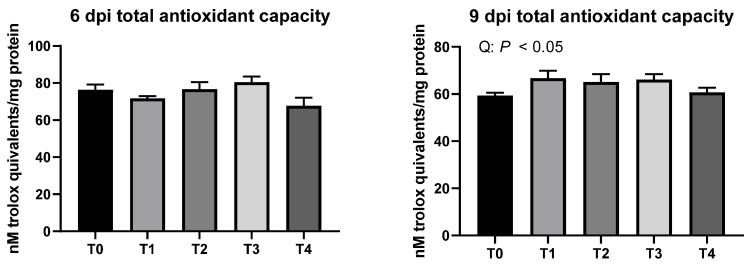
Total antioxidant capacity in the liver of broiler chickens infected with different dosages of *Eimeria tenella*. On 6 and 9 days post-infection (dpi), total antioxidant capacity in the liver was measured in the T0 (treatment 0; Sham-challenged with phosphate-buffered saline); T1 (treatment 1; challenged with 6250 sporulated oocysts of *E. tenella*); T2 (treatment 2; challenged with 12,500 sporulated oocysts of *E. tenella*); T3 (treatment 3; challenged with 25,000 sporulated oocysts of *E. tenella*); T4 (treatment 4; challenged with 50,000 sporulated oocysts of *E. tenella*) groups. At each time point, orthogonal polynomial contrasts analysis was conducted to see linear pattern (L) and quadratic pattern (Q) among the treatments.

As shown in Figure 6, liver TAC was not modulated due to *E. tenella* infection in broiler chickens (*p* > 0.1) on 6 dpi. However, different inoculation dosages of *E. tenella* quadratically increased liver TAC on 9 dpi (*p* < 0.05).

### 3.6. VFA Concentrations in Cecal Digesta

As shown in Table 6, infection of *E. tenella* linearly decreased acetate concentration in ceca contents (*p* < 0.05), and the T4 group had significantly lower acetate concentration in ceca contents compared to the T0 group. Isobutyrate concentration in ceca contents was linearly decreased (*p* < 0.05) on 6 dpi as *E. tenella* dosage increased. Total VFA were linearly decreased due to *E. tenella* infection (*p* < 0.05), and the T4 group had significantly lower total VFA compared to the T0 group on 6 dpi. On 9 dpi, *E. tenella* tended to linearly decrease butyrate (*p* = 0.09), and significantly decreased valerate concentrations (Linear; *p* < 0.05) in ceca contents. The T0 group had significantly lower propionate compared to the T1 group, and T4 birds had significantly lower varelate compared to the T1 group.

## 4. Discussion

The purpose of the study was to investigate the effects of different inoculation dosages of *E. tenella* on growth performance, gastrointestinal permeability, oocyst shedding, intestinal morphology, fecal consistency, ileal apparent digestibility, antioxidant capacity, and cecal VFA profile in broiler chickens. Inoculation dosages were derived from our previous study [19], and the same strain of *E. tenella* was used. However, in the current study, milder infection (lesion score of T4: 1.6) was achieved compared to our previous study (lesion score of the T4 equivalent group: 2.7). The potential reason for milder infection would be that broilers were challenged with three different *Eimeria* spp. (*E. acervulina*, *E. maxima,* and *E. tenella*) in the previous study, which probably caused more severe infection by compensating the immune system in birds compared to the single species challenging in the current study. Mild-infection models are important to test a new bioactive compound because if the compounds do not show any beneficial effects at the mild infection model, they may not show any beneficial effects against coccidiosis at severe infection model either. In the present study, *E. tenella* infection decreased feed efficiency during the acute phase and increased feed intake on 6 and 7 dpi. These data are partially consistent with previous studies [24,25] which reported that FCR was increased along with reduced BW of broilers. In our current study, however, *E. tenella* infection increased FCR by increasing the feed intake of birds, whereas BW was also numerically (*p* = 0.16) decreased with a linear trend during the acute phase. Impaired feed efficiency in broilers infected with *E. tenella* during the acute phase in the present study would be associated with reduced acetate and total VFA production in the ceca. Acetate is the most abundant VFA, which are produced via bacterial fermentation in the ceca of broilers [26]. It is already well-established that *E. tenella* infection can negatively affect cecal microbiome, which results in depressed VFA production in broilers according to many previous studies [27,28]. Cecal VFA production affects the host’s energy balance because VFA are important energy substrates for the host [29]. Gasaway [30] mentioned that VFA supply approximately 11% to 18% of the total energy production in chickens. In addition, *Eimeria* spp. compete for energy and nutrients for their asexual and sexual replications with the host [31]. Reduced available energy in the body can result in reduced BW or increased FCR by raising feed intake of the birds to decrease maintenance energy requirements or to meet the energy requirements, respectively [32]. In addition, reduced production of acetate, which can induce the secretion of satiety-stimulating hormones from the gut, could increase the feed intake of the birds [33]. Therefore, in this study, reduced VFA production in ceca due to *E. tenella* infection potentially reduced available energy levels in the body, and this increased feed intake and FCR to supply more energy to meet the energy requirements in broiler chickens infected with *E. tenella*.

This study showed that *E. tenella* infection deepened crypts depth of the ceca. The potential reasons for increased cecal crypts depth due to *E. tenella* infection are still unclear. It is proposed that *E. tenella* increased cecal CD (mucosa layer) to make their habitats in the ceca, or ceca crypts were deepened to increase VFA absorption because VFA production was restricted due to *E. tenella* infection. Nevertheless, deepened CD could inhibit the production and absorption of VFA in the ceca. Increased CD possibly turned in increased total goblet cells in the ceca and mucus secretion into the cecal contents. This possibly reduced concentration of VFA and modulated VFA production in ceca content by affecting microbiome of the broiler chickens. Ceca only have villi at the entrance of the ceca to filter large particles away and to act as an immunological detector of cecal contents [34,35], and middle and distal parts of the ceca only have smooth mucous membrane without villi [36]. Thereby, another possible reason for increased CD in broilers infected with *E. tenella* would be that birds increased CD to let crypts function like villi as a defensive mechanism in the proximal ceca because *E. tenella* infected ceca are vulnerable for further infections (e.g., bacterial infection).

We also hypothesized that impaired ceca health due to *E. tenella* infection may indirectly affect small intestinal health (the main area for nutrient digestion and absorption) by causing energy deficiency and inducing oxidative stress, and this may account for decreased feed efficiency in the current study. Nevertheless, no differences were observed in DM, OM, and ash apparent ileal digestibility among the treatments on 6 and 9 dpi in the current study. According to our previous study, *E. maxima* infection significantly decreased nutrient digestibility in broiler chickens, which indicates that different *Eimeria* spp. affect nutrient digestibility of broilers differently [5]. Liver total antioxidant capacity was same among the treatments on 6 dpi and even increased in broilers infected with *E. tenella* on 9 dpi, potentially because mild infection of *E. tenella* may allow birds to stimulate their antioxidant defensive system. Still, over-production of antioxidants (enzymatic and non-enzymatic) can result in over-use of energy and nutrients, which also may decrease available energy and nutrients for growth in chickens. However, a previous study by Georgieva et al. [25] reported that severe *E. tenella* infection model decreased antioxidant capacity of broilers. Ileal crypt depth was decreased, and ileal VH:CD was increased without affecting VH in *E. tenella* infected broilers in the current study. Probably, ileal morphology was enhanced with limited energy and nutrients sources as a compensation mechanism because cecal functionality was restricted due to *E. tenella* in the current study. There were negative effects of *E. tenella* on duodenal and jejunal morphology on 9 dpi and gastrointestinal permeability on 7 dpi. Energy and absorbed VFA play an important role in gut development in broiler chickens by stimulating gut epithelial cell proliferation [9]. In this study, reduced cecal VFA production would be the main factor that negatively affected gut health of *E. tenella* infected broilers rather than increased pathogens and toxin production because liver health was maintained in broilers infected with *E. tenella.* Whereas it cannot be concluded that impaired intestinal health caused impaired feed efficiency during the acute phase, energy deficiency due to poor VFA yield subsequently damaged intestinal health of broilers infected with *E. tenella*. Moreover, while differences were observed in gastrointestinal permeability, it was only less than two-fold difference. Our previous study [37] reported that more than 200 folds differences of gastrointestinal permeability were observed due to *E. maxima* infection. Hence, reduced VFA production in the ceca due to *E. tenella* infection caused energy deficiency in the body, which resulted in compensated gut health in broiler chickens.

In this study, different *E. tenella* inoculation dosages linearly increased oocyst shedding on 5 to 6 dpi, which implies that the higher inoculation dosages can results in higher oocyst shedding. Nonetheless, these data could be obtained in our study because our *E. tenella* strain induced mild-infection (lesion score below 2) in the ceca. Williams [38] demonstrated that higher inoculation dosage levels can linearly increase oocyst yields until reaching to the crowded dosages (e.g., maximally producing dosage), and higher dosages than the crowded dosages can decrease oocyst yields in broilers. However, no differences were observed on 6 to 7 dpi and 7 to 8 dpi in the current study. The current result also demonstrated that different challenge dosages of *E. tenella* have different peak point of shedding. The potential reason would be that higher number of *E. tenella* possibly decreased number of generation within the asexual and sexual stages of the life [35], which resulted in an earlier peak date for the highest dosage group in the study. Our study was the first to find that different *E. tenella* inoculation dosages resulted in different peak points for oocyst yields in the mildly infected broilers. Oocyst yields in severely infected (*E. tenella* lesion score 3 to 4) broilers at different time points should be investigated further. 

Ileal and fecal moisture contents were modulated due to Eimeria infection in the current study. Ileal (6 dpi) and fecal moisture contents (5 to 6 dpi) were quadratically and linearly decreased, respectively. Afterwards, fecal moisture content was increased on 6 to 8 dpi. *E. tenella* are known to induce diarrhea containing mucus and blood. Although obvious bloody diarrhea was not achieved in the current study, fecal moisture content was increased after 6 dpi. Potentially, *E. tenella* damaged enterocytes in the ceca and modulated microbiota, and this caused electrolyte imbalance in the ceca which may explain the modulated ileal and fecal moisture contents in the current study [39]. Moreover, ceca play a crucial role in water absorption [40], and water absorption ability of the ceca of birds infected with *E. tenella* would be limited potentially due to thickened mucosa layer in the current study. Hypothetically, higher water loss due to *E. tenella* infection increased water intake [40] as birds increased their feed intake to compensate energy deficiency in the current study. In addition, increased fecal moisture content can result in increased litter moisture, and this can increase the incidence of food pad dermatitis in broilers [41]. 

This current study showed that mild infection of *E. tenella* impaired feed efficiency and gut health mainly through reducing VFA production in broilers. Thereby, supplementation of VFA or bioactive compounds that has high energy values and antimicrobial effects (e.g., medium chain fatty acids) can control mild-infection of *E. tenella* in broilers [11,42].

## 5. Conclusions

Orthogonal polynomial contrasts showed that *E. tenella* mild-infection reduced VFA production in the ceca, and this caused energy deficiency, which increased feed intake and impaired feed efficiency of broiler chickens. This suggests that the cecal VFA concentrations could be a key parameter to represent feed efficiency and *E. tenella* infection severity in broiler chickens. Furthermore, mild-infection of *E. tenella* modulated intestinal morphology, antioxidant capacity, and gastrointestinal permeability in the recovery phase. Different inoculation dosages of *E. tenella* changed oocyst shedding patterns and ileal/fecal moisture content. These current data showed that the mechanisms of *E. tenella* impair feed efficiency and gut health of broilers, which will be beneficial to study strategies to cope with *E. tenella* infection in broiler chickens. Based on the current study, 25,000 to 50,000 sporulated oocyst dosage range would be recommended as subclinical models for nutritional strategies.

## Figures and Tables

**Table 1:** Diet composition and calculated analysis of the broiler diet (g/kg, as fed basis).

Ingredients	D 14 to 23
Corn	700.8
Soybean meal (480 g crude protein/kg)	241.73
Soybean oil	15.84
Deflour phosphate	13.99
Sand	7.00
Limestone	6.11
Titanium dioxide	3.00
DL-Methionine 99%	2.86
L-Lysine HCl 78%	2.80
Vitamin Premix ^1^	2.50
Common Salt	1.79
L-threonine	0.77
Mineral Premix ^2^	0.80
Total	1,000
Calculated energy and nutrient value, %	
Metabolizable energy, Mcal/kg	3,100
Crude protein	18.375
SID ^3^ Methionine	0.552
SID Total sulfur amino acids	0.8
SID Lysine	1.02
SID Threonine	0.66
Total calcium	0.76
Available phosphate	0.38

^1^ Vitamin mix provided the following in mg/100 g diet: thiamine-HCl, 1.5; riboflavin 1.5; nicotinic acid amide 15; folic acid 7.5; pyridoxine-HCl, 1.2; d-biotin 3; vitamin B-12 (source concentration, 0.1%) 2; d-calcium pantothenate 4; menadione sodium bisulfite, 1.98; α-tocopherol acetate (source 500,000 IU/g), 22.8; cholecalciferol (source 5,000,000 IU/g) 0.09; retinyl palmitate (source 500,000 IU/g), 2.8; ethoxyquin, 13.34; I-inositol, 2.5; dextrose, 762.2; ^2^ Mineral mix provided the following in g/100 g diet: Ca(H_2_PO_4_)_2_·H_2_O, 3.62; CaCO_3_, 1.48; KH_2_PO_4_, 1.00; Na_2_SeO_4_, 0.0002; MnSO_4_·H_2_O, 0.035; FeSO_4_·7H_2_O, 0.05; MgSO_4_·7H_2_O, 0.62; KIO_3_, 0.001; NaCl, 0.60; CuSO_4_·5H_2_O, 0.008; ZnCO_3_, 0.015; CoCl_2_·6H_2_O, 0.00032; NaMoO_4_·2H_2_O, 0.0011; KCl, 0.10; dextrose, 0.40; ^3^ SID: standard ileal digestible amino acid.

**Table 2:** Growth performance parameters including body weight (BW; g), average daily gain (ADG; g/d), average daily feed intake (ADFI; g/d), and feed conversion ratio (FCR; g/g) of broiler chickens infected with different dosages of *Eimeria tenella* during the acute phase [0 to 6 days post-infection (dpi)] and recovery phase (6 to 9 dpi) ^1^.

Items		*Eimeria tenella*-Challenged						Polynomial Contrast	
	T0	T1	T2	T3	T4	SEM	*p* Value	Lin.	Quad.
Initial BW	359.4	360.3	356	359.5	358	3.72	0.38		
0 to 6 dpi									
BW	703.4	705.5	697	699.5	680.77	27.28	0.55	0.16	0.43
ADG	57.33	57.53	57.01	56.54	53.80	4.32	0.56	0.16	0.37
ADFI	84.92	87.24	88.63	86.05	84.31	4.61	0.55	0.69	0.11
FCR	1.48	1.52	1.55	1.52	1.57	0.06	0.19	0.04	0.64
6 to 9 dpi									
BW	881.8	859	876.9	885.73	889.19	79.63	0.97	0.69	0.72
ADG	65.81	57.40	61.68	63.83	69.70	13.74	0.62	0.43	0.22
ADFI	115.4	120.9	123.77	123.1	123.1	14.86	0.87	0.4	0.55
FCR	1.9	2.17	2.03	1.97	1.77	0.39	0.50	0.38	0.16
0 to 9 dpi									
ADG	60.16	57.49	58.83	58.97	59.10	6.47	0.97	0.94	0.66
ADFI	95.09	98.47	100.6	98.16	97.24	6.81	0.72	0.65	0.22
FCR	1.62	1.74	1.71	1.67	1.64	0.14	0.54	0.85	0.15

^1^ T0: treatment 0 (Sham-challenged with phosphate-buffered saline); T1, treatment 1 (challenged with 6250 sporulated oocysts of *Eimeria tenella*); T2, treatment 2 (challenged with 12,500 sporulated oocysts of *Eimeria tenella*); T3, treatment 3 (challenged with 25,000 sporulated oocysts of *Eimeria tenella*); T4, treatment 4 (challenged with 50,000 sporulated oocysts of *Eimeria tenella*). At each time point, orthogonal polynomial contrasts analysis was conducted to see linear pattern (L) and quadratic pattern (Q) among the treatments.

**Table 3:** Fecal moisture content (fecal consistency) and ileal moisture content of broiler chickens infected with different dosages of *Eimeria tenella on* 5 to 6 days post-infection (dpi), 6 to 7 dpi, and 7 to 8 dpi (fecal moisture content), and at 6 and 9 dpi (ileal moisture content) ^1^.

Items		*Eimeria tenella*-Challenged						Polynomial Contrast	
	T0	T1	T2	T3	T4	SEM	*p* Value	Lin.	Quad.
Feces									
5 to 6 dpi	78.23	76.76	75.91	76.56	76.99	1.66	0.22	0.22	0.04
6 to 7 dpi	68.14	69.68	73.38	74.64	68.66	4.87	0.12	0.35	0.03
7 to 8 dpi	70.81	71.87	73.09	75.07	74.86	2.52	0.03	<0.01	0.65
Ileum									
6 dpi	81.75	81.83	80.6	80.94	80.81	0.76	0.03	0.01	0.33
9 dpi	80.34	80.1	81.24	80.62	79.93	1.22	0.42	0.9	0.18

^1^ T0: treatment 0 (Sham-challenged with phosphate-buffered saline); T1, treatment 1 (challenged with 6250 sporulated oocysts of *E. tenella*); T2, treatment 2 (challenged with 12,500 sporulated oocysts of *E. tenella*); T3, treatment 3 (challenged with 25,000 sporulated oocysts of *E. tenella*); T4, treatment 4 (challenged with 50,000 sporulated oocysts of *E. tenella*). At each time point, orthogonal polynomial contrasts analysis was conducted to see linear pattern (L) and quadratic pattern (Q) among the treatments.

**Table 4:** Duodenal, jejunal, ileal, and cecal morphology [villus height (VH; μm), crypts depth (CD; μm), and VH:CD] of broiler chickens infected with different dosages of *Eimeria tenella* on 6 days post-infection (dpi) and 9 dpi ^1^.

Items		*Eimeria tenella*-Challenged						Polynomial Contrast	
	T0	T1	T2	T3	T4	SEM	*p* Value	Lin.	Quad.
6 dpi									
Duodenum									
VH	2459.97	2585.52	2442.42	2154.12	2485.42	300.16	0.22	0.34	0.56
CD	282.46	245.17	253.82	227.51	255.55	35.36	0.17	0.13	0.08
VH:CD	9.05	10.8	9.86	10.39	9.85	1.78	0.53	0.61	0.26
Jejunum									
VH	1411.46	1375.62	1332.65	1321.93	1303.21	147	0.71	0.17	0.76
CD	234.92	222.03	223.93	221.29	225.43	34.52	34.52	0.66	0.57
VH:CD	6.38	6.56	6.08	6.28	5.94	1.22	0.91	0.47	0.85
Ileum									
VH	899.01	936.51	934.37	894.06	879.21	113.9	0.87	0.58	0.41
CD	194.11	181.49	183.02	184.97	161.46	24.03	0.24	0.06	0.59
VH:CD	4.92	5.28	5.18	5.09	5.72	0.59	0.22	0.08	0.54
Cecal CD	217.21 ^b^	410.22 ^a^	451.86 ^a^	593.18 ^a^	477.05 ^a^	111.85	<0.01	<0.01	<0.01
9 dpi									
Duodenum									
VH	2475	2574.81	2452.6	2150.15	2375.54	278.63	0.17	0.1	0.87
CD	250.69	226.78	233.48	211.26	234.56	35.58	0.49	0.31	0.23
VH:CD	10.26	11.55	11.04	10.74	10.32	1.95	0.77	0.78	0.28
Jejunum									
VH	1410.12	1348.81	1320.96	1288.44	1257.51	159.32	0.54	0.09	0.82
CD	208.29	217.18	197.49	211.8	210.85	34.22	0.87	0.99	0.78
VH:CD	7.21	6.55	7.06	6.5	6.21	1.86	0.69	0.26	0.87
Ileum									
VH	888.68	917.4	879.2	885.43	876.32	108.22	0.97	0.69	0.85
CD	173.99	167.73	158.63	161.9	155.46	28.8	0.8	0.26	0.78
VH:CD	5.35	5.57	5.74	5.7	5.95	0.72	0.69	0.17	0.89
Cecal CD	87.57 ^b^	163.55 ^a,b^	182.47 ^a^	224.19 ^a^	196.3 ^a^	47.71	<0.01	<0.01	0.02

^1^ T0: treatment 0 (Sham-challenged with phosphate-buffered saline); T1, treatment 1 (challenged with 6250 sporulated oocysts of *E. tenella*); T2, treatment 2 (challenged with 12,500 sporulated oocysts of *E. tenella*); T3, treatment 3 (challenged with 25,000 sporulated oocysts of *E. tenella*); T4, treatment 4 (challenged with 50,000 sporulated oocysts of *E. tenella*). At each time point, orthogonal polynomial contrasts analysis was conducted to see linear pattern (L) and quadratic pattern (Q) among the treatments. Different letters at the same time point represent significantly different (*p* < 0.05) by proc mixed followed by the Tukey’s multiple comparison test among the treatment groups.

**Table 5:** Apparent ileal digestibility (%) of dry matter (DM), organic matter (OM), and ash of broiler chickens infected with different dosages of *Eimeria tenella* on 6 days post-infection (dpi) and 9 dpi.

Items		*Eimeria tenella*-Challenged ^1^						Polynomial Contrast	
	T0	T1	T2	T3	T4	SEM	*p* Value	Lin.	Quad.
6 dpi									
DM	69.16	70.31	68.35	71.53	70.19	1.96	0.09	0.21	0.96
OM	70.85	72.08	70.13	73.32	71.79	1.94	0.09	0.23	0.89
Ash	40.16	40	37.66	40.82	42.66	5.47	0.6	0.42	0.26
9 dpi									
DM	72.71	70.31	71.86	72.44	73.5	2.8	0.42	0.36	0.2
OM	75.02	72.29	74.32	74.68	75.8	2.69	0.29	0.31	0.18
Ash	33.03	36.24	29.47	33.86	33.82	9.49	0.81	0.95	0.76

^1^ T0: treatment 0 (Sham-challenged with phosphate-buffered saline); T1, treatment 1 (challenged with 6250 sporulated oocysts of *E. tenella*); T2, treatment 2 (challenged with 12,500 sporulated oocysts of *E. tenella*); T3, treatment 3 (challenged with 25,000 sporulated oocysts of *E. tenella*); T4, treatment 4 (challenged with 50,000 sporulated oocysts of *E. tenella*). At each time point, orthogonal polynomial contrasts analysis was conducted to see linear pattern (L) and quadratic pattern (Q) among the treatments.

**Table 6:** Concentrations of volatile fatty acids (mM) in ceca contents of broiler chickens infected with different dosages of *Eimeria tenella* at 6 days post-infection (dpi) and 9 dpi ^1^.

Items		*Eimeria tenella*-Challenged ^1^						Polynomial Contrast	
	T0	T1	T2	T3	T4	SEM	*p* Value	Lin.	Quad.
6 dpi									
Acetate	48.21 ^a^	35.2 ^a,b^	35.64 ^a,b^	36.94 ^a,b^	26.91 ^b^	10	0.02	<0.01	0.65
Propionate	8.37	9.03	0.84	7.27	6.1	2.95	0.5	0.11	0.45
Isobutyrate	0.26	0.17	0.19	0.15	0.12	0.08	0.07	<0.01	00.73
Butyrate	8.69	5.8	7.4	5.34	4.96	3.8	0.42	0.12	0.82
Isovalerate	0.42	0.42	0.45	0.32	0.37	0.17	0.72	0.35	0.82
Valerate	1.09	0.95	0.78	0.69	0.8	0.51	0.72	0.22	0.47
Total VFA	67.04 ^a^	51.58 ^a,b^	52.52 ^a,b^	50.73 ^a,b^	39.26 ^b^	13.95	0.04	<0.01	0.8
9 dpi									
Acetate	50.77	76.52	62.01	46.47	48.11	27.04	0.31	0.32	0.25
Propionate	6.14 ^b^	11.87 ^a^	8.17 ^a,b^	7.82 ^a,b^	7.07 ^a,b^	3.34	0.06	0.62	0.07
Isobutyrate	0.16	0.32	0.15	0.16	0.16	0.14	0.22	0.39	0.55
Butyrate	8.64	13.04	8.58	6.24	6.3	4.94	0.16	0.09	0.4
Isovalerate	0.26	0.53	0.39	0.37	0.6	0.39	0.59	0.31	0.96
Valerate	0.96 ^a,b^	1.65 ^a^	0.86 ^a,b^	0.8 ^a,b^	0.57 ^b^	0.55	0.03	0.03	0.21
Total VFA	66.94	103.94	68.72	61.86	62.83	35.97	0.26	0.29	0.43

^1^ T0: treatment 0 (Sham-challenged with phosphate-buffered saline); T1, treatment 1 (challenged with 6250 sporulated oocysts of *E. tenella*); T2, treatment 2 (challenged with 12,500 sporulated oocysts of *E. tenella*); T3, treatment 3 (challenged with 25,000 sporulated oocysts of *E. tenella*); T4, treatment 4 (challenged with 50,000 sporulated oocysts of *E. tenella*). At each time point, orthogonal polynomial contrasts analysis was conducted to see linear pattern (L) and quadratic pattern (Q) among the treatments. Different letters at the same time point represent significantly different (*p* < 0.05) by proc mixed followed by the Tukey’s multiple comparison test among the treatment groups.

## Data Availability

The data presented in this study are available on request from the corresponding author. The data are not publicly available due to further statistical analysis.

## References

[1] Blake D.P., Knox J., Dehaeck B., Huntington B., Rathinam T., Ravipati V., Ayoade S., Gilbert W., Adebambo A.O., Jatau I.D. (2020). Re-calculating the cost of coccidiosis in chickens. Vet. Res..

[2] Choi J., Kim W.K. (2020). Dietary application of tannins as a potential mitigation strategy for current challenges in poultry production: A review. Animals.

[3] Vermeulen A., Schaap D., Schetters T.P. (2001). Control of coccidiosis in chickens by vaccination. Vet. Parasitol..

[4] López-Osorio S., Chaparro-Gutiérrez J.J., Gómez-Osorio L.M. (2020). Overview of poultry Eimeria life cycle and host-parasite interactions. Front. Vet. Sci..

[5] Teng P.-Y., Yadav S., Shi H., Kim W.K. (2021). Evaluating endogenous loss and standard ileal digestibility of amino acids in response to the graded severity levels of E. maxima infection. Poult. Sci..

[6] Gaboriaud P., Sadrin G., Guitton E., Fort G., Niepceron A., Lallier N., Rossignol C., Larcher T., Sausset A., Guabiraba R. (2020). The absence of gut microbiota alters the development of the apicomplexan parasite Eimeria tenella. Front. Cell. Infect. Microbiol..

[7] Zaman M.A., Iqbal Z., Abbas R.Z., Khan M.N. (2012). Anticoccidial activity of herbal complex in broiler chickens challenged with Eimeria tenella. Parasitology.

[8] Cason J., Hinton A., Northcutt J., Buhr R., Ingram K., Smith D., Cox N. (2007). Partitioning of external and internal bacteria carried by broiler chickens before processing. J. Food Prot..

[9] Shang Y., Kumar S., Oakley B., Kim W.K. (2018). Chicken gut microbiota: Importance and detection technology. Front. Vet. Sci..

[10] Józefiak D., Rutkowski A., Martin S. (2004). Carbohydrate fermentation in the avian ceca: A review. Anim. Feed Sci. Technol..

[11] Zhang J.-M., Sun Y.-S., Zhao L.-Q., Chen T.-T., Fan M.-N., Jiao H.-C., Zhao J.-P., Wang X.-J., Li F.-C., Li H.-F. (2019). SCFAs-induced GLP-1 secretion links the regulation of gut microbiome on hepatic lipogenesis in chickens. Front. Microbiol..

[12] Broom L.J. (2021). Evidence-based consideration of dietary ‘alternatives’ to anticoccidial drugs to help control poultry coccidial infections. Worlds Poult. Sci. J..

[13] Noack S., Chapman H.D., Selzer P.M. (2019). Anticoccidial drugs of the livestock industry. Parasitol. Res..

[14] Blake D.P., Tomley F.M. (2014). Securing poultry production from the ever-present Eimeria challenge. Trends Parasitol..

[15] Giannenas I., Florou-Paneri P., Papazahariadou M., Christaki E., Botsoglou N., Spais A. (2003). Effect of dietary supplementation with oregano essential oil on performance of broilers after experimental infection with Eimeria tenella. Arch. Anim. Nutr..

[16] Memon F., Yang Y., Lv F., Soliman A., Chen Y., Sun J., Wang Y., Zhang G., Li Z., Xu B. (2021). Effects of probiotic and Bidens pilosa on the performance and gut health of chicken during induced Eimeria tenella infection. J. Appl. Microbiol..

[17] Zhou Z., Nie K., Huang Q., Li K., Sun Y., Zhou R., Wang Z., Hu S. (2017). Changes of cecal microflora in chickens following Eimeria tenella challenge and regulating effect of coated sodium butyrate. Exp. Parasitol..

[18] Song X., Li Y., Chen S., Jia R., Huang Y., Zou Y., Li L., Zhao X., Yin Z. (2020). Anticoccidial effect of herbal powder “Shi Ying Zi” in chickens infected with Eimeria tenella. Animals.

[19] Teng P.-Y., Yadav S., de Souza Castro F.L., Tompkins Y.H., Fuller A.L., Kim W.K. (2020). Graded Eimeria challenge linearly regulated growth performance, dynamic change of gastrointestinal permeability, apparent ileal digestibility, intestinal morphology, and tight junctions of broiler chickens. Poult. Sci..

[20] Johnson J., Reid W.M. (1970). Anticoccidial drugs: Lesion scoring techniques in battery and floor-pen experiments with chickens. Exp. Parasitol..

[21] Short F., Gorton P., Wiseman J., Boorman K. (1996). Determination of titanium dioxide added as an inert marker in chicken digestibility studies. Anim. Feed Sci. Technol..

[22] Lin Y., Olukosi O.A. (2021). Qualitative and quantitative profiles of jejunal oligosaccharides and cecal short-chain fatty acids in broiler chickens receiving different dietary levels of fiber, protein and exogenous enzymes. J. Sci. Food Agric..

[23] Lourenco J.M., Kieran T.J., Seidel D.S., Glenn T.C., Silveira M.F.D., Callaway T.R., Stewart R.L. (2020). Comparison of the ruminal and fecal microbiotas in beef calves supplemented or not with concentrate. PLoS ONE.

[24] Christaki E., Florou-Paneri P., Giannenas I., Papazahariadou M., Botsoglou N.A., Spais A.B. (2004). Effect of a mixture of herbal extracts on broiler chickens infected with Eimeria tenella. Anim. Res..

[25] Georgieva N., Koinarski V., Gadjeva V. (2006). Antioxidant status during the course of Eimeria tenella infection in broiler chickens. Vet. J..

[26] Aljumaah M.R., Alkhulaifi M.M., Abudabos A.M., Alabdullatifb A., El-Mubarak A.H., Al Suliman A.R., Stanley D. (2020). Organic acid blend supplementation increases butyrate and acetate production in Salmonella enterica serovar Typhimurium challenged broilers. PLoS ONE.

[27] Macdonald S.E., Nolan M.J., Harman K., Boulton K., Hume D.A., Tomley F.M., Stabler R.A., Blake D.P. (2017). Effects of Eimeria tenella infection on chicken caecal microbiome diversity, exploring variation associated with severity of pathology. PLoS ONE.

[28] Kley M.-V., Oviedo-Rondón E., Dowd S., Hume M., Nalian A. (2012). Effect of Eimeria infection on cecal microbiome of broilers fed essential oils. ARS USDA Submiss..

[29] LeBlanc J.G., Chain F., Martín R., Bermúdez-Humarán L.G., Courau S., Langella P. (2017). Beneficial effects on host energy metabolism of short-chain fatty acids and vitamins produced by commensal and probiotic bacteria. Microb. Cell. Fact..

[30] Gasaway W.C. (1976). Volatile fatty acids and metabolizable energy derived from cecal fermentation in the willow ptarmigan. Comp. Biochem. Physiol. Part A Physiol..

[31] Santos T.S.D., Teng P.-Y., Yadav S., Castro F.L.D.S., Gould R.L., Craig S.W., Chen C., Fuller A.L., Pazdro R., Sartori J.R. (2020). Effects of Inorganic Zn and Cu Supplementation on Gut Health in Broiler Chickens Challenged with *Eimeria* spp.. Front. Vet. Sci..

[32] Renaudeau D., Gilbert H., Noblet J. (2012). Effect of climatic environment on feed efficiency in swine. Feed Efficiency in Swine.

[33] Canfora E.E., Blaak E.E. (2017). Acetate: A diet-derived key metabolite in energy metabolism: Good or bad in context of obesity and glucose homeostasis?. Curr. Opin. Clin. Nutr. Metab. Care.

[34] Adji A.V., Plumeriastuti H., Ma’ruf A., Legowo D. (2019). Histopathological Alterations of Ceca in Broiler Chickens (*Gallus gallus*) Exposed to Chronic Heat Stress. World.

[35] Walker R.A., Sharman P.A., Miller C.M., Lippuner C., Okoniewski M., Eichenberger R.M., Ramakrishnan C., Brossier F., Deplazes P., Hehl A.B. (2015). RNA Seq analysis of the Eimeria tenella gametocyte transcriptome reveals clues about the molecular basis for sexual reproduction and oocyst biogenesis. BMC Genom..

[36] Clench M.H., Mathias J.R. (1995). The avian cecum: A review. Wilson Bull..

[37] Teng P.-Y., Choi J., Tompkins Y., Lillehoj H., Kim W. (2021). Impacts of increasing challenge with Eimeria maxima on the growth performance and gene expression of biomarkers associated with intestinal integrity and nutrient transporters. Vet. Res..

[38] Williams R. (2001). Quantification of the crowding effect during infections with the seven Eimeria species of the domesticated fowl: Its importance for experimental designs and the production of oocyst stocks. Int. J. Parasitol..

[39] Chadwick E., Rahimi S., Grimes J., Pitts J., Beckstead R. (2020). Sodium bisulfate feed additive aids broilers in growth and intestinal health during a coccidiosis challenge. Poult. Sci..

[40] Nakahiro Y., Isshiki Y. (1975). Effect of cecal ligation on digestibility of crude fiber, cellulose and pentosan in chickens. Jpn. Poult. Sci..

[41] Van der Hoeven-Hangoor E., Van de Linde I., Paton N., Verstegen M., Hendriks W. (2013). Effect of different magnesium sources on digesta and excreta moisture content and production performance in broiler chickens. Poult. Sci..

[42] Faradilla Z.S., Yunus M., Hermadi H.A. (2020). The Effect of Dietary Administration of Virgin Coconut oil on Differential Leukocytes in Infected Chicken with Eimeria tenella. J. World’s Poult. Res..

